# Dihydroisotanshinone I as a Treatment Option for Head and Neck Squamous Cell Carcinomas

**DOI:** 10.3390/ijms22168881

**Published:** 2021-08-18

**Authors:** Cheng-Ming Hsu, Ming-Yu Yang, Ming-Shao Tsai, Geng-He Chang, Yao-Hsu Yang, Yao-Te Tsai, Ching-Yuan Wu, Shun-Fu Chang

**Affiliations:** 1 Department of Otolaryngology-Head and Neck Surgery, Chiayi Chang Gung Memorial Hospital, Chiayi 61363, Taiwan; scm0031@cgmh.org.tw (C.-M.H.); b87401061@cgmh.org.tw (M.-S.T.); a9244@cgmh.org.tw (G.-H.C.); yaote1215@gmail.com (Y.-T.T.); 2 School of Medicine, College of Medicine, Chang Gung University, Taoyuan 33302, Taiwan; 3 Graduate Institute of Clinical Medical Sciences, College of Medicine, Chang Gung University, Taoyuan 33302, Taiwan; yangmy@mail.cgu.edu.tw; 4 Department of Otolaryngology, Kaohsiung Chang Gung Memorial Hospital, Kaohsiung 83301, Taiwan; 5 Department of Chinese Medicine, Chiayi Chang Gung Memorial Hospital, Chiayi 61363, Taiwan; r95841012@cgmh.org.tw; 6 School of Chinese Medicine, College of Medicine, Chang Gung University, Taoyuan 33302, Taiwan; 7 Department of Medical Research and Development, Chiayi Chang Gung Memorial Hospital, Chiayi 61363, Taiwan

**Keywords:** head and neck squamous cell carcinoma, danshen, *Salvia miltiorrhiza*, dihydroisotanshinone I, apoptosis, p38 signaling

## Abstract

Head and neck squamous cell carcinomas (HNSCCs) are the most common cancers of the head and neck, and their prevalence is rapidly increasing. HNSCCs present a clinical challenge because of their high recurrence rate, therapeutic resistance to radiation and chemotherapy drugs, and adverse effects. Hence, traditional Chinese herbal treatment may be advantageous to therapeutic strategies for HNSCCs. Danshen (*Salvia miltiorrhiza*), a well-known Chinese herb, has been extensively applied in treatments for various diseases, including cancer, because of its high degree of safety and low rate of adverse effects despite its unclear mechanism. Thus, we aimed to explore the possible anticancer effects and mechanisms of dihydroisotanshinone I (DT), a compound in danshen (extract from danshen), on HNSCCs. Three HNSCCs cell lines were used for in vitro studies, and a Detroit 562 xenograft mouse model was chosen for in vivo studies. Our in vitro results showed that DT could initiate apoptosis, resulting in cell death, and the p38 signaling partially regulated DT-initiated cell apoptosis in the Detroit 562 model. In the xenograft mouse model, DT reduced tumor size with no obvious adverse effect of hepatotoxicity. The present study suggests that DT is a promising novel candidate for anti-HNSCCs therapy.

## 1. Introduction

Head and neck squamous cell carcinomas (HNSCCs) are the most common cancers of the head and neck and mainly occur in the epithelial mucosa of the oral cavity, pharynx, and larynx [1,2]. The risk factors for HNSCCs include tobacco and alcohol use, environmental pollutants, and viral infections [2]. The prevalence of HNSCCs is increasing rapidly, and the incidence of HNSCCs is anticipated to rise by 30% by 2030. HNSCCs are the top ten cancers worldwide according to several epidemiological studies [3,4,5]; HNSCCs are also the fifth leading cause of cancer death in Taiwan [6].

In clinical practice, the therapeutic strategy for HNSCCs comprise surgical resection with chemotherapy or adjuvant radiation [7,8,9,10]. However, it is not uncommon that tumor recurrence and adverse response occur especially in patients with recurrent HNSCCs [2]. Hence, a greater understanding of the molecular mechanisms of HNSCCs progression and further development of new therapeutic strategies, such as traditional Chinese herbal treatment, warrant further investigation.

Danshen (*Salvia miltiorrhiza*), an important traditional Chinese herb, has been used extensively as a healthy food and in clinical usage for more than 1000 years [11,12,13]. Modern advanced technological analysis has clarified that the major constituents of danshen are hydrophilic phenolic acids and lipophilic compounds [11,12,13]. Studies have shown that both these constituents contribute to most of the bioactivities and therapeutic capabilities of danshen, including antioxidation [13], anti-inflammation [14], and mitigation of cardiovascular diseases [15]. In the past two decades, researchers had more interest in the aspects of cancer therapy in danshen. Tanshinone, one of the lipophilic compounds of danshen, has been identified as a major factor influencing cancer cell survival [12,13]. Many in vitro and in vivo studies have demonstrated the anticancer effects of danshen/tanshinone in different types of cancers, including liver cancer [16], colorectal cancer [17], breast cancer [18], lung cancer [19], ovarian cancer [20], pancreatic cancer [21], and gastric cancers [22]. Tanshinone has been classified into four types according to its structural diversity: tanshinone IIA (Tan IIA), cryptotanshinone (CT), tanshinone I (Tan I), and dihydroisotanshinone I (DT) [12,13]. Among these, Tan IIA is the most studied and well-known in terms of clinical application and cancer treatment [23], whereas DT is the least studied. The anticancer effects and mechanisms of DT have been insufficiently elucidated in the past; thus, it is encouraging to investigate this intensively.

Because the clinical application of traditional Chinese herbal medicine in treating HNSCCs has yielded positive results, the present study proposed that danshen may be a potential candidate for HNSCCs therapy. Thus, this study aimed to examine the possible anticancer effects of DT on HNSCCs and the underlying mechanisms of these effects.

## 2. Results

### 2.1. DT Inhibited Cell Survival of Human HNSCCs Cells

**Figure 1:**
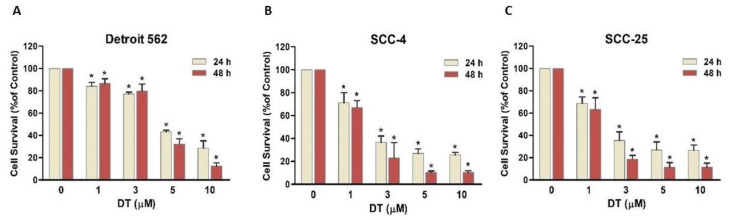
Dihydroisotanshinone I (DT) inhibited survival of HNSCCs cells in a dose- and time-dependent manner. The inhibitory effect of DT on (**A**) Detroit 562, (**B**) SCC-4, and (**C**) SCC-25 cells was assessed using MTT assay after 24- and 48-hours treatments with 1-, 3-, 5-, and 10-µM DT. Data presented are the means and standard errors of the mean of three independent experiments. * indicates a statistically significant difference compared with untreated control cells of the same treatment duration.

To investigate whether DT inhibits the survival of HNSCCs cells, three HNSCCs cell lines, namely, Detroit 562 (pharynx carcinoma), SCC-4 (tongue carcinoma), and SCC-25 (tongue carcinoma), were treated with DT (1, 3, 5, and 10 µM) for 24 and 48 h, and survival rates were assessed by mitochondrial conversion of the 3-(4,5-dimethylthiazol-2-yl)-2,5-diphenyltetrazolium bromide (MTT) assay. As shown in Figure 1, DT inhibited the cell survival of all three HNSCCs cell lines in a dose- and time-dependent manner. In the Detroit 562 cells, DT concentrations of 5 and 10 µM resulted in a larger decrease in cell survival (Figure 1A). In the SCC-4 and SCC-25 cells, DT concentrations of 3 µM and higher inhibited cell survival (Figure 1B,C).

### 2.2. DT-Induced Apoptosis in HNSCCs Cells

**Figure 2:**
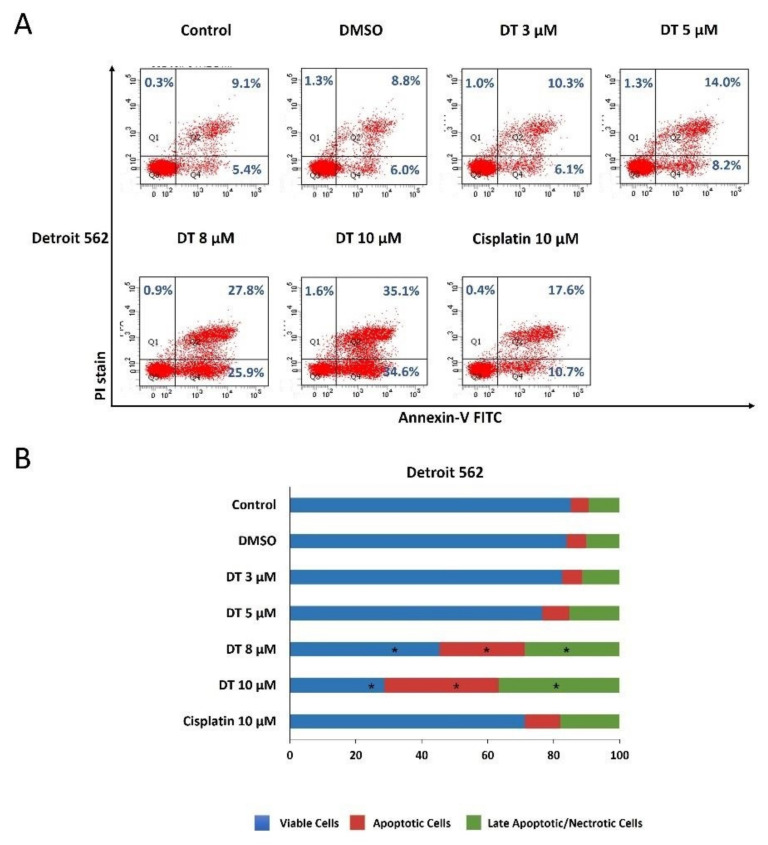
DT-induced apoptosis in Detroit 562 cells. (**A**) Detroit 562 cells were treated with DMSO or DT (3, 5, 8, and 10 µM) for 24 h, and the percentages of viable cells (annexin V(−)/PI(−)), apoptotic cells (annexin V(+)/PI(−), and late apoptotic/necrotic cells (annexin V(−)/PI(+) and annexin V(+)/PI(+)) were determined through annexin V/PI staining followed by flow cytometric analysis. (**B**) The distribution of viable, apoptotic, and late apoptotic/necrotic cells after DT treatment was calculated from the means from three independent experiments. * indicates a statistically significant difference compared with untreated control cells.

**Figure 3:**
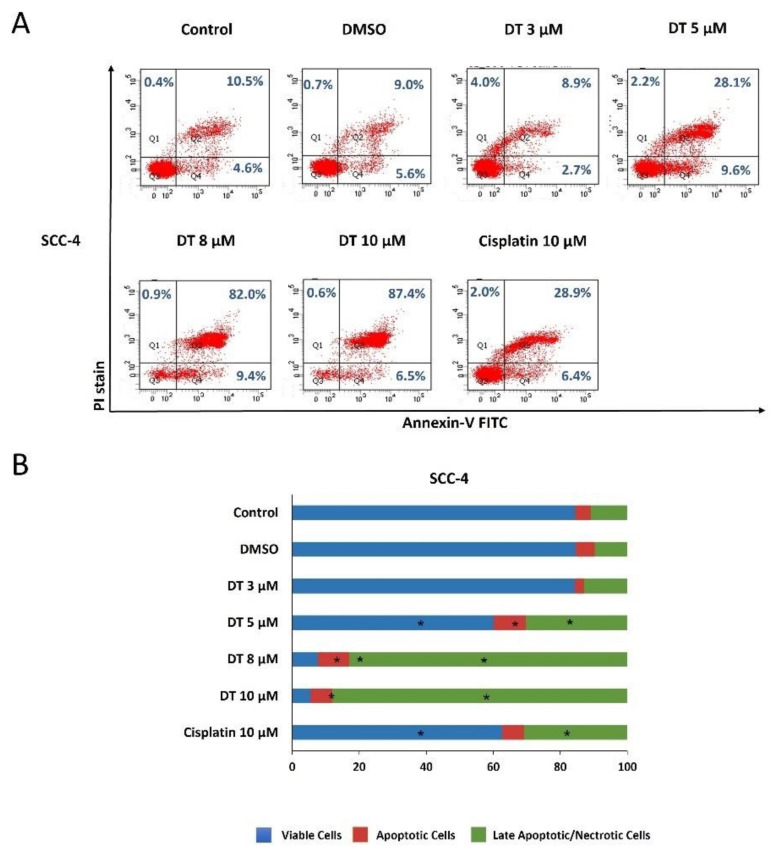
DT-induced apoptosis in SCC-4 cells. (**A**) SCC-4 cells were treated with DMSO or DT (3, 5, 8, and 10 µM) for 24 h, and the percentages of viable cells (annexin V(−)/PI(−)), apoptotic cells (annexin V(+)/PI(−), and late apoptotic/necrotic cells (annexin V(−)/PI(+) and annexin V(+)/PI(+)) were determined through annexin V/PI staining followed by flow cytometric analysis. (**B**) The distribution of viable, apoptotic, and late apoptotic/necrotic cells after DT treatment was calculated from the means from three independent experiments. * indicates a statistically significant difference compared with untreated control cells.

**Figure 4:**
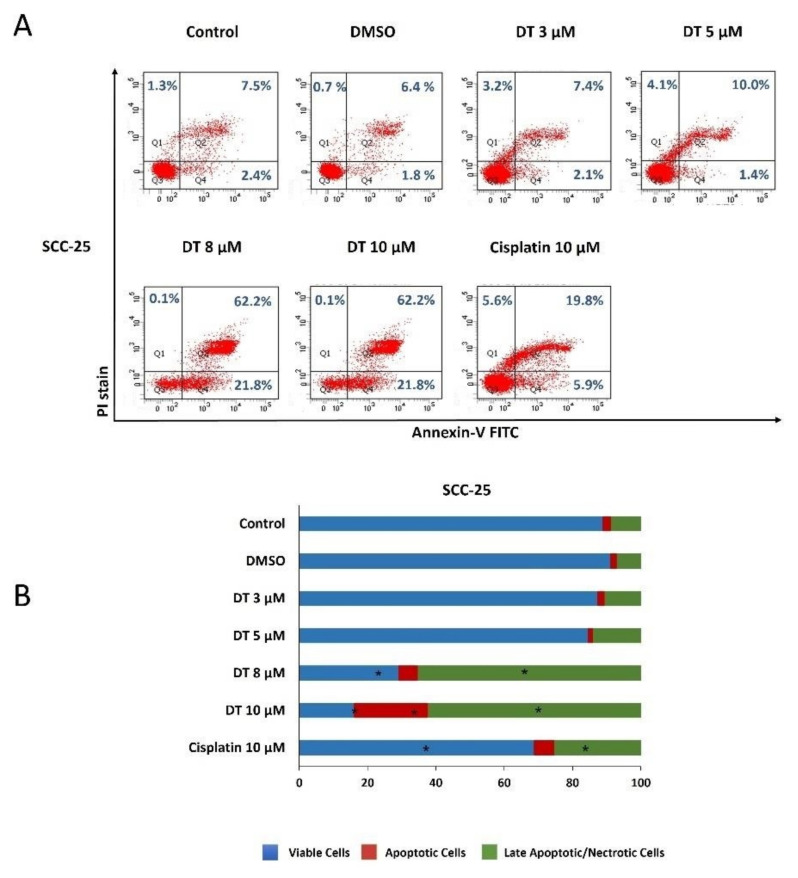
DT-induced apoptosis in SCC-25 cells. (**A**) SCC-25 cells were treated with DMSO or DT (3, 5, 8, and 10 µM) for 24 h, and the percentages of viable cells (annexin V(−)/PI(−)), apoptotic cells (annexin V(+)/PI(−), and late apoptotic/necrotic cells (annexin V(−)/PI(+) and annexin V(+)/PI(+)) were determined through annexin V/PI staining followed by flow cytometric analysis. (**B**) The distribution of viable, apoptotic, and late apoptotic/necrotic cells after DT treatment was calculated from the means from three independent experiments. * indicates a statistically significant difference compared with untreated control cells.

To examine if DT could induce apoptosis of HNSCCs cells, Detroit 562, SCC-4, and SCC-25 cells were kept as controls or treated with dimethyl sulfoxide (DMSO) or DT (3, 5, 8, and 10 µM) for 24 h and analyzed through propidium iodide (PI) and annexin V–fluorescein isothiocyanate (FITC) double-staining using flow cytometry. All three HNSCCs cells lines treated with 8- and 10-µM DT demonstrated significant reductions in live cells and increases in apoptotic and late apoptotic/necrotic cells, and this effect was time- and dose-dependent (Figure 2A, Figure 3A and Figure 4A). Moreover, the cell-killing effect of DT appeared more effective in SCC-4 and SCC-25 cells than in Detroit 562 cells (Figure 2B, Figure 3B and Figure 4B). The apoptosis-inducing ability of 10-µM DT was better than 10-µM cisplatin.

### 2.3. DT Increased Expression of Caspase-3 and Caspase-8 in HNSCCs Cells

**Figure 5:**
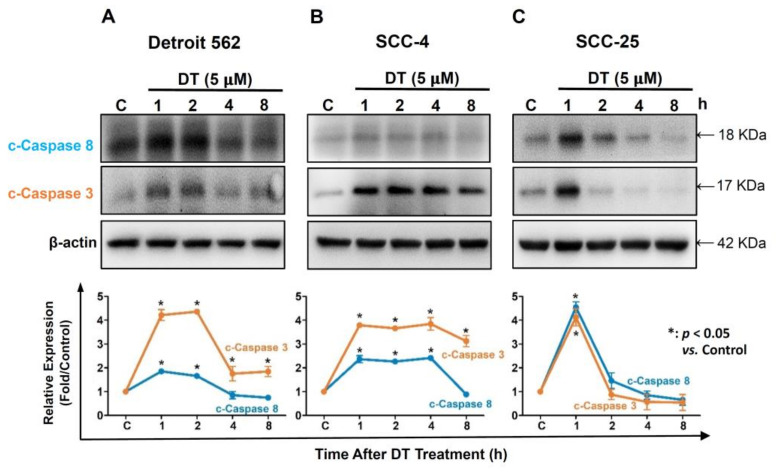
DT induced the cleavage of caspase-8 and caspase-3 in HNSCCs cells. Cleaved forms of caspase-8 and caspase-3 were increased in (**A**) Detroit 562, (**B**) SCC-4, and (**C**) SCC-25 cells after treatment with 5-µM DT as determined by Western blotting. The fold changes were calculated from three independent experiments and are presented as means and standard errors of the mean. * indicates a statistically significant difference compared with untreated control cells.

Caspases were identified as the controllers of apoptosis progression. Hence, we investigated the effect of DT on the expression of cleaved caspase-3 and caspase-8 in the three HNSCCs cell lines. Cells were treated with 5-µM DT for 1, 2, 4, and 8 h, and the expression of cleaved caspase-3 and caspase-8 was analyzed through Western blotting. As shown in Figure 3, the expression of cleaved caspase-3 and caspase-8 significantly increased in all three HNSCCs cells lines treated with 5-µM DT. The levels of both caspases increased greatly after 1 h of DT treatment, followed by a decline after 4 h in Detroit 562 cells (Figure 5A), 8 h in SCC-4 cells (Figure 5B), and 2 h in SCC-25 cells (Figure 5C).

### 2.4. p38 Signaling Regulated DT-Induced Apoptosis in Detroit 562 Cells

**Figure 6:**
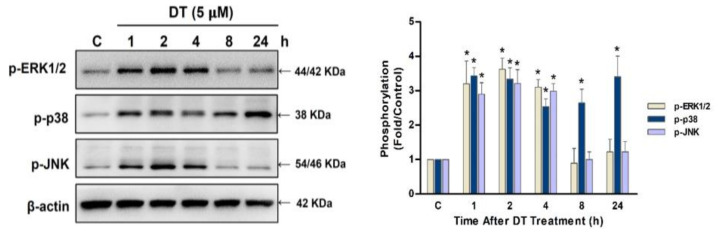
DT induced the phosphorylation of ERK1/2, p38, and JNK kinases in Detroit 562 cells. Cells were treated with 5-µM DT for 1, 2, 4, 8, and 24 h, and antibodies against phosphorylated (p)-ERK1/2, p-p38, and p-JNK were used to analyze the phosphorylation status of these kinases through Western blotting. The fold changes were calculated from three independent experiments and are presented as means and standard errors of the mean. * indicates a statistically significant difference compared with untreated control cells.

To further elucidate the signaling pathway associated with DT-induced apoptosis, Detroit 562, SCC-4, and SCC-25 cells were treated with 5- or 10-µM DT for 1, 2, 4, 8, and 24 h to examine the phosphorylation of ERK1/2, p38, and JNK kinases. The phosphorylation of ERK1/2, p38, and JNK kinases increased significantly after 1 h of DT treatment (Figure 4). The phosphorylation of ERK1/2 and JNK kinases declined after 8 h of DT treatment, but the phosphorylation of p38 persisted for 24 h (Figure 6).

**Figure 7:**
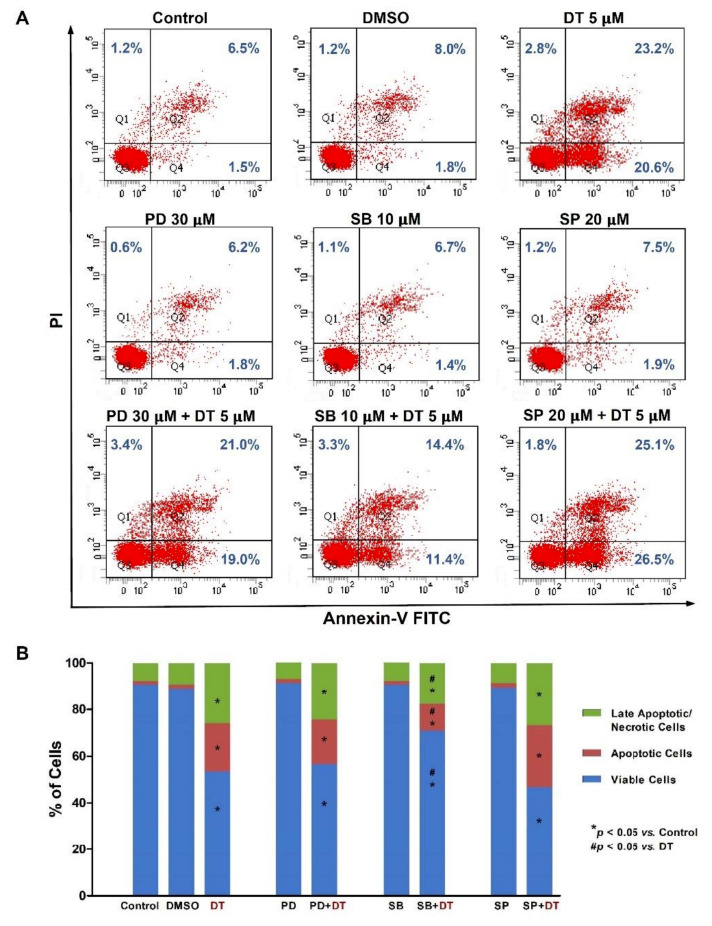
p38 Signaling regulated DT-induced apoptosis in Detroit 562 cells. (**A**) Cells were pretreated with specific kinase inhibitors of ERK1/2 (PD98059, 30 μM), p38 (SB203580, 10 μM), or JNK (SP600125, 20 μM) for 1 h and then treated with 5-µM DT for 24 h; the percentages of viable cells, apoptotic cells, and late apoptotic/necrotic cells were determined by annexin V/PI staining followed by flow cytometric analysis. (**B**) The distributions of viable, apoptotic, and late apoptotic/necrotic cells after treatment were calculated from the means from three independent experiments. * and # indicate statistically significant differences compared with untreated control cells and 5-µM DT-treated cells, respectively.

To further validate that DT-induced apoptosis was regulated by the DT-induced phosphorylation of ERK1/2, p38, and JNK kinases, we pretreated Detroit 562 cells with specific inhibitors of ERK1/2 (PD98059, 30 μM), p38 (SB203580, 10 μM), or JNK (SP600125, 20 μM) for 1 h and then treated the cells with 5-µM DT for 48 h. Flow cytometric analysis showed that DT-induced apoptosis could be partially recovered by the inhibition of p38 kinase activity, but not by the inhibition of ERK1/2 or JNK kinases in Detroit 562 cells (Figure 7). DT-induced apoptosis could not be recovered by inhibition of p38 kinase activity or ERK1/2 or JNK kinases in SCC-4 and SCC-25.

### 2.5. DT Suppressed Detroit 562 Tumor Growth in BALB/cAnN.Cg Nude Mice

**Figure 8:**
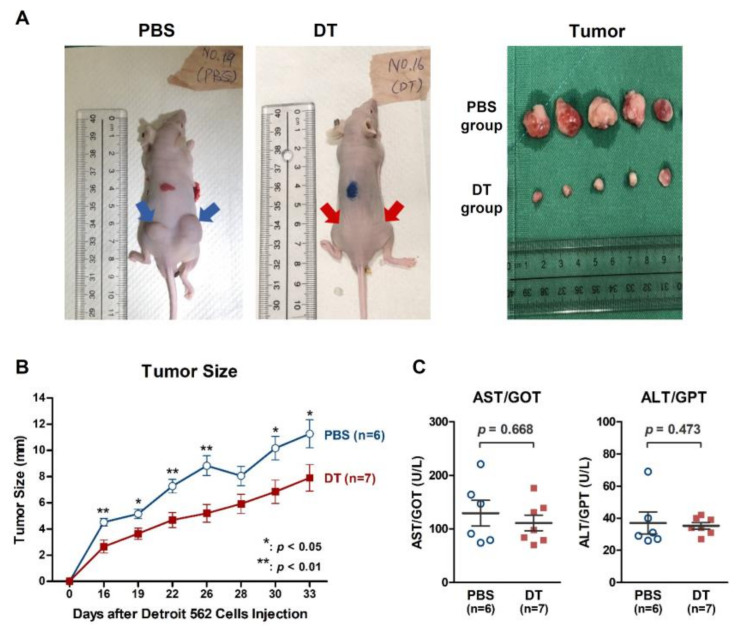
Antitumor effect of DT in the Detroit 562 HNSCCs xenograft model. (**A**) DT suppressed Detroit 562 tumor growth in xenografted mice. (**B**) The average tumor size in the DT group was significantly smaller than that in the control group. (**C**) Serum AST/GOT and ALT/GPT levels did not reveal a significant difference in liver function between the PBS and DT treatment groups. * indicates a statistically significant difference between the DT group and the PBS group.

To examine the exact in vivo antitumor effects of DT, we established a xenograft model by subcutaneously injecting Detroit 562 cells into nude mice (BALB/cAnN.Cg) to induce the growth of xenograft tumors. These Detroit 562-xenografted mice were then treated with intraperitoneal injections of phosphate-buffered saline (PBS) for controls or DT (30 mg/kg) over 33 consecutive days. Significantly greater tumor regression occurred in the DT-treated mice than in the control mice (Figure 8A). The size of the tumors in the DT-treated group was reduced by nearly 20% compared with that in the control group (Figure 8B). We also examined the plasma AST/GOT and ALT/GPT levels (Figure 8C) and found that DT treatment did not exert significant hepatotoxicity in the mice.

## 3. Discussion

As far as we know, no previous studies have investigated the anticancer effects of the well-known traditional Chinese herb danshen in HNSCCs. In this study, we examined the ability of DT, a key tanshinone in danshen, to inhibit the survival and growth of HNSCCs in Detroit 562 (pharynx carcinoma) and SCC-4/SCC-25 (tongue carcinoma) cells as well as in a xenograft mouse model. The systematic experiments in the present study revealed that (1) DT significantly promoted cell death in the three HNSCCs cell lines; (2) DT induced the cleavage of caspase-3 and caspase-8, and consequently initiated apoptosis in the three HNSCCs cell lines; (3) p38 signaling partially regulated DT-induced apoptosis in Detroit 562 cells; and (4) DT effectively reduced the tumor size in Detroit 562-xenografted mice and did not elicit markedly liver inflammation.

SCC-4 and SCC-25 cells were more sensitive to DT treatment than Detroit-562 cells. The apoptosis-inducing effect of DT was more dominant in SCC-4 and SCC-25 cells than in Detroit 562 cells. The DT-induced apoptosis could be partially recovered by the inhibition of p38 kinase activity only in Detroit 562 cells but not in SCC-4 and SCC-25. Therefore, the mechanism underlying the cytotoxic effect of DT in SCC-4 and SCC-25 may be different from that in Detroit 562 cells. The p38 kinase signaling may not play a role in DT-induced cell death of SCC-4 and SCC-25 cells. The difference may have resulted from some genomic variation or mutation, such as mutated PIK3CA in Detroit 562 cell lines but not in SCC-25 [24].

Traditional Chinese herbal medicine has received increasing attention. The reason is that Chinese herbs are derived from plants with evidences of high safety and less adverse effects [25,26]. Danshen has been used as a healthy food and as medicine for approximately 1000 years. Originally, danshen acted in cardiovascular and cerebrovascular diseases because it facilitates blood circulation and stasis clearance [27,28]. However, accumulating evidences have declaimed the additional efficacy of danshen in treating many types of cancer by its blockage of cancer growth and metastasis [18,20,22,29,30,31,32,33,34,35,36]. In support of these pre-existing studies, our data reveal similar benefits of danshen for HNSCCs, cancers for which danshen’s therapeutic effects have not been extensively investigated. Our results demonstrated that DT, an important lipophilic compound in danshen, significantly increased apoptosis and induced cell death in three HNSCCs cell lines. Moreover, DT blocked tumor growth in the Detroit 562 xenograft mouse model. HNSCCs indeed demonstrate clinical challenges owing to their high recurrence rate and resistance to therapeutic radiation and chemotherapy drugs, such as platinum-based agents (cisplatin and 5-fluorouracil) [2,37,38]. Furthermore, clinical observations have demonstrated that patients with HNSCCs who undergo chemotherapy with platinum-based drugs typically experience adverse effects, such as nephrotoxicity and hepatopathy, which may lead to greater difficulty in curing the disease [2,37,38]. Our results stated that DT’s anticancer treatment in Detroit 562-xenografted mice did not influence serum AST and ALT levels, which can be used to assess liver damage and hepatotoxicity [39]. This finding is notable because it implies that DT is a potential substitute for chemotherapy drugs and can reduce the occurrence of vital organ damage. Hence, our study suggests that the traditional Chinese herbal treatment with danshen is a promising candidate for an alternative choice of anticancer strategies in patients with HNSCCs.

Although less research has been conducted using DT compared with other tanshinones, DT has attracted growing attention [12]. The anticancer effects of DT were reported in researches involving liver cancer cells [16], colon cancer cells [40], and osteosarcoma cells [41]; these effects appear to occur through the initiation of apoptosis and cell death, as well as the inhibition of proliferation, angiogenesis, and metastasis in cancer cells. Moreover, the latest findings have shown a protective effect of DT in patients with breast cancer [18] and prostate cancer [42]. Mitogen-activated protein kinase cascade pathways, such as ERK1/2, p38, and JNK signaling, have been extensively studied for their regulation of normal cell function and anticancer mechanisms. Among these pathways, p38 signaling was considered to be involved in the anticancer function by testing four types of tanshinones [12]. However, in our study, although DT activated ERK1/2, p38, and JNK signaling in HNSCCs cells, only p38 signaling could partially regulate the effect of DT on apoptosis in HNSCCs cells. Therefore, according to our study, we propose that the therapeutic mechanism of DT in HNSCCs is complex and warrants further elucidation. The therapeutic strategies for HNSCCs include surgery with adjuvant chemoradiotherapy, primary chemoradiotherapy, or systemic immunotherapy. DT could be a treatment option for HNSCCs.

In conclusion, this in vitro and in vivo study revealed that the danshen extract DT reduced the survival of HNSCCs cells. Our results confirmed the contribution of DT-induced apoptosis in the in vitro HNSCCs cell growth through blocking p38 signaling. DT also significantly reduced tumor size in xenograft nude mice without hepatotoxicity. These results suggest that DT could be a novel anti-HNSCCs agent, although further prospective randomized studies are needed to validate these results.

## 4. Materials and Methods

### 4.1. Cell Culture

Three human HNSCCs cell lines, namely, Detroit 562, SCC-4, and SCC-25, were purchased from the Taiwan Food Industry Research and Development Institute, Hsinchu, Taiwan (R.O.C.). Detroit 562 cells are pharyngeal squamous cell carcinoma cells derived from pleural effusion. SCC-4 and SCC-25 cells are tongue squamous cell carcinoma cells. All the cells were grown in a minimum essential medium (MEM)-F12 medium (Invitrogen, Life Technologies, Carlsbad, CA, USA) containing 0.4 µg/mL hydrocortisone (Sigma-Aldrich, St. Louis, MO, USA) and 10% fetal bovine serum at 37 °C in an atmosphere of 5% CO_2_.

### 4.2. MTT Assay

Three human HNSCCs cell lines were treated with various concentrations of DT, and the percentages of metabolically active cells were determined based on the reduction in MTT to formazan. Briefly, after treatment, the culture medium was replaced with MEM (without phenol) containing 0.02% MTT (Sigma-Aldrich, St. Louis, MO, USA) and then incubated for 4 h. Finally, the medium was replaced with 200 μL of DMSO, and the absorbance was read at 570 nm using a 96 well–format DTX880 multimode detector (Beckman Coulter, Brea, CA, USA). The background absorbance was measured in wells containing only the dye solution and culture medium. Absorbance values were calculated by subtracting the background values, and all experiments were performed in triplicate.

### 4.3. Western Blotting

Samples were extracted in RIPA buffer (20 mM of Tris-HCl at pH 7.5, 150 mM of NaCl, 1 mM of Na_2_EDTA, 1 mM of EGTA, 1% NP-40, 1% sodium deoxycholate, 2.5 mM of sodium pyrophosphate, 1 mM of β-glycerophosphate, 1 mM of Na3VO4, and 1 μg/mL of leupeptin). For Western blotting, 30 μg of total lysates was separated through 6–15% sodium dodecyl sulfate-polyacrylamide gel electrophoresis and transferred to a PVDF membrane (Millipore, Darmstadt, Germany). After being blocked with nonfat dry milk for 1 h, the membranes were incubated overnight with primary antibodies at 1:3000 dilution. Primary antibodies against phosphorylated epitopes and cleaved caspase-3 and caspase-8 (all purchased from Cell Signaling Technologies, Danvers, MA, USA). β-Actin (1:5000 dilution, Sigma-Aldrich, Saint Louis, MO, USA) was used as an internal control. The secondary antibodies were horseradish peroxidase (HRP)-conjugated goat anti-mouse IgG (Sigma-Aldrich) and goat anti-rabbit IgG (Sigma-Aldrich). The membranes were briefly incubated with Western Lightning Plus-ECL enhanced chemiluminescence substrate (PerkinElmer Inc., Waltham, MA, USA) to visualize the proteins.

### 4.4. Annexin V–FITC/PI Staining for Flow Cytometry

Three human HNSCCs cell lines were seeded in a 100-mm plate and cultured overnight before treatment. After treatment, the apoptotic and necrotic cells were detected by annexin V binding and PI uptake by using an Annexin V–FITC Apoptosis Detection Kit (Becton Dickinson), according to the manufacturer’s instructions. Samples were analyzed using flow cytometry (BD Bioscience FacsCanto II Flow Cytometer, Marshall Scientific, Hampton, NH, USA).

### 4.5. Mouse Xenograft Model

All procedures involving animals were approved by the Animal Care and Use Committee of Chang Gung Memorial Hospital (approval number 2015060201). Thirteen male BALB/cAnN.Cg nude mice weighing 18 to 20 g and aged 5 to 7 weeks were obtained from BioLASCO Taiwan Co., Ltd. to establish the xenograft model. Detroit 562 cells were subcutaneously injected (1 × 10^6^ cells per mouse) into the left and right flanks of the mice. After 2 days, the mice were randomized into two groups (6 and 7 mice) and treated with PBS (*n* = 6) or 30 mg/kg DT (*n* = 7) intraperitoneally every 2 days. Tumor size and body weight were measured every 3–5 days for 5 weeks. Tumor size, body weight, and mortality of the mice were monitored daily. After 5 weeks, the mice were euthanized.

### 4.6. Statistical Analyses

All values are presented as means ± standard errors of the mean of the replicate samples, and experiments were performed three times. Differences between the two groups were assessed using unpaired two-tailed Student’s *t*-tests. *p* values < 0.05 were considered statistically significant. All the statistical analyses in this study were performed using SPSS version 15.0 (SPSS, Chicago, IL, USA).

## Data Availability

All data generated or analyzed during this study are included in this published article.
